# Retentive Force Assessment and Wear Exploration of CAD-CAM Fabricated PEEK, Zirconia, and PMMA Mini Ball Attachments for Tooth-Supported Overdenture: A Laboratory Study

**DOI:** 10.3390/dj14080490

**Published:** 2026-08-06

**Authors:** Abdullah Kamel, James Kit Hon Tsoi, Haytham Mohsen

**Affiliations:** 1 Department of Prosthetic Dentistry, Faculty of Dentistry, Minia University, Minia 61519, El-Minia Governorate, Egypt; haitham.mohsen@mu.edu.eg; 2 Dental Materials Science, Applied Oral Sciences and Community Dental Care, Faculty of Dentistry, The University of Hong Kong, Hong Kong SAR, China

**Keywords:** computer-aided design, dental prosthesis retention, dental abutments, dental materials, zirconium, polyetheretherketone, polymethyl methacrylate, denture, overlay

## Abstract

**Background/Objectives**: The longevity of tooth-supported overdentures depends on the retentive stability of attachment systems, yet limited evidence exists on the performance of non-metallic CAD-CAM fabricated mini ball attachments. This study aimed to evaluate the retentive force changes and surface wear of CAD-CAM fabricated mini ball attachments made of zirconia, polyetheretherketone (PEEK), and polymethyl methacrylate (PMMA) for tooth-supported overdentures. **Methods**: Twenty-one mandibular canine replicas received CAD-CAM copings with mini ball attachments (n = 7 per material). Retentive force was measured at baseline and after 90, 270, 540, 1080 and 2160 insertion–removal cycles (simulating 2 years of clinical use). Surface wear was examined under scanning electron microscopy (one specimen per group, preliminary). Data were analyzed using one-way ANOVA, paired *t*-tests with Bonferroni correction, and mixed repeated-measures ANOVA (Greenhouse–Geisser correction). Effect sizes (partial η^2^, Cohen’s d) were calculated. Based on a priori power analysis 7 specimens per group were decided. **Results**: All materials showed a significant decline in retention over cycles (*p* < 0.001, partial η^2^ = 0.90). No significant differences were found among the three materials at any time point (*p* > 0.05). The material × time interaction was not significant (*p* = 0.064). Within each material, baseline-to-final reductions were significant (*p* ≤ 0.009, Cohen’s d > 3.0). Exploratory SEM suggested minimal wear for zirconia, moderate for PEEK, and pronounced surface deterioration for PMMA. **Conclusions**: Within the limitations of this study, all three CAD-CAM mini ball attachments provided decreasing but measurable retention over simulated use. Zirconia showed numerically the highest final retention, but no statistically significant differences were detected among materials. Clinically, zirconia attachments demonstrated numerically superior final retention with minimal wear, suggesting potential advantages for long-term use; however, the lack of statistically significant differences among materials and the preliminary nature of the wear analysis warrant caution, emphasizing the need for future clinical validation before definitive material recommendations can be made.

## 1. Introduction

Tooth-supported overdentures are a well-established, effective treatment modality for geriatric patients who have retained only a few natural teeth. Retaining natural abutments preserves alveolar bone, enhances proprioception, improves prosthesis stability, and provides an economical alternative to implant-retained prostheses [1,2].

The preservation of remaining natural teeth through overdenture therapy has gained increasing acceptance in contemporary prosthodontic practice. This treatment modality offers significant advantages over conventional complete dentures, including enhanced masticatory efficiency, improved stability, and better patient satisfaction [3]. Moreover, the maintenance of periodontal ligament mechanoreceptors in retained roots provides proprioceptive feedback that is lost with implant-supported prostheses, contributing to improved neuromuscular control and occlusal perception [4]. Retention and stability of overdentures can be further enhanced by attachment systems, which can be categorized based on whether the abutments are splinted or not. When the abutments are splinted, the linkage between the prosthesis and the abutments is achieved using bar structures. If the dental abutments are not splinted, each abutment connects individually to the prosthetic framework. This connection can be made using systems such as Locator, ERA, ball, O-ring, and different magnetic systems [3,4].

The Locator and ball attachments are preferred because of their simplicity in application, reduced technical demands, and lower costs when compared to bar or magnet systems. Ball attachment is commonly utilized for both root-supported and implant-supported prostheses. It consists of a metal ball fixed to the abutment tooth, while a matrix component is attached to the prosthetic device, designed to fit over the ball and held in place by a frictional mechanism [5,6]. The selection of appropriate attachment systems represents a critical decision in overdenture treatment planning. Attachment systems are broadly classified into splinted (bar-type) and unsplinted (individual) designs, each with distinct biomechanical and clinical implications. Bar attachments provide rigid splinting of abutments, offering enhanced stability and load distribution but demanding greater technical complexity, cost, and hygiene maintenance challenges [6]. Conversely, unsplinted attachments such as Locator, ERA, ball, and O-ring systems offer simpler fabrication, easier cleaning access, and reduced patient costs [7,8]. Among these, ball attachments remain particularly popular due to their simplicity, predictable retention characteristics, and compatibility with both tooth and implant abutments. The ball attachment system consists of a metallic ball component affixed to the abutment and a nylon matrix housed within the prosthesis, providing retention through frictional engagement [9]. Due to their advantageous physical and mechanical characteristics, metal alloys are often utilized in dental prosthetics. However, galvanic corrosion can occur, especially when these alloys contact different metals in the oral cavity [7]. In addition, milling metal using CAD/CAM is demanding and causes severe abrasion of the milling burs [8]. Consequently, a different material alternative to metal for the construction of attachments using CAD/CAM systems is in demand.

While traditional ball attachments are fabricated from precious or non-precious metal alloys such as gold-platinum or titanium, these materials present several clinical and technical challenges. Galvanic corrosion can occur when dissimilar metals coexist in the oral environment, potentially compromising attachment integrity and causing patient discomfort [10,11]. Additionally, the high hardness of metal alloys imposes significant wear on milling burs during CAD/CAM fabrication, increasing production costs and reducing manufacturing efficiency [12]. These limitations have driven the search for alternative materials that combine favorable mechanical properties with biocompatibility, esthetics, and CAD/CAM compatibility.

Zirconia (zirconium dioxide) is now recognized as a promising biomaterial in prosthodontics, widely used for crowns, bridges, implant abutments, and telescopic crowns due to its high flexural strength (900–1200 MPa), hardness (~1200 HV), and transformation-toughening mechanism, which enhances resistance to crack propagation. Its low surface roughness and good biocompatibility also contribute to patient satisfaction [8,9,10]. PEEK is a high-performance polymer with excellent biocompatibility, shock absorption, and a favorable modulus of elasticity similar to bone. Its machinability makes it suitable for CAD/CAM frameworks and attachments [11]. PMMA, a conventional denture base material, has been adapted into CAD/CAM fabrication, offering improved polymerization, reduced residual monomer release, and better surface properties compared with conventionally processed PMMA [12]. However, its relatively low hardness raises concerns about long-term wear resistance when used in attachment components.

The selection of zirconia, PEEK, and PMMA for this investigation was guided by their distinct material properties and clinical relevance. Zirconia was selected for its established high wear resistance and mechanical strength, making it a candidate for long-term attachment applications despite limited data on ball attachments specifically. PEEK was chosen due to its favorable modulus of elasticity (similar to bone), which may facilitate stress distribution, and its increasing clinical adoption in CAD/CAM frameworks. PMMA was included as a representative of conventional polymer materials adapted to CAD/CAM technology, offering a cost-effective baseline for comparison despite its lower hardness. Together, these materials represent a spectrum of mechanical properties, allowing assessment of how material characteristics influence retentive performance.

Although zirconia, PEEK, and PMMA have been individually studied in various prosthodontic applications, little is known about their performance in CAD/CAM-fabricated ball attachments. Therefore, the present study aimed to compare the retentive force and wear patterns of zirconia, PEEK, and PMMA mini ball attachments during simulated clinical aging. Current study tested two null hypotheses: (1) there would be no significant decrease in retentive force after 2160 insertion removal cycles compared to baseline; and (2) the type of material (zirconia, PEEK, PMMA) would not significantly affect retentive force or wear characteristics.

## 2. Methods

Three test groups were established to examine retentive force and explore surface wear. A total of 21 CAD/CAM constructed mini ball attachments, made of zirconia, polyetheretherketone (PEEK), and polymethyl methacrylate (PMMA), were tested in this study. Retention force over 6 time period is simulating 2 years of function. In addition, surface wear was explored by scanning electron microscope (SEM).

### 2.1. Sample Size Estimation

To determine the minimum number of specimens required for this three-arm laboratory study (PEEK, zirconia, PMMA), we carried out an a priori power analysis using G*Power software (version 3.1.9.7). The effect size was derived from a previous investigation by Kamel A. and colleagues [13], which reported retentive forces after 2000 cycles: PEEK secondary crowns on titanium abutments (mean 13.0 N, SD = 7.9) and zirconia secondary crowns (mean 2.9 N, SD = 2.6). For a one way ANOVA with three groups, a significance level α of 0.05, and a statistical power of 85%, the required total sample size was calculated to be 15 specimens (5 per group). Because adding a third material (PMMA) might increase variability and to guard against possible technical losses during cyclic testing, we choose 7 specimens per group (total n = 21). This sample size provides more than adequate power (well above 85%) to detect clinically meaningful differences in retention among the three tested materials, assuming effect sizes comparable to those reported in the literature.

The 21 specimens were allocated by simple randomization into three groups of seven specimens each, corresponding to the three tested materials. Randomization was performed using a computer-generated random number sequence (Excel, Microsoft Corp.) to ensure unbiased distribution.

### 2.2. Specimen Preparation

**Figure 1:**
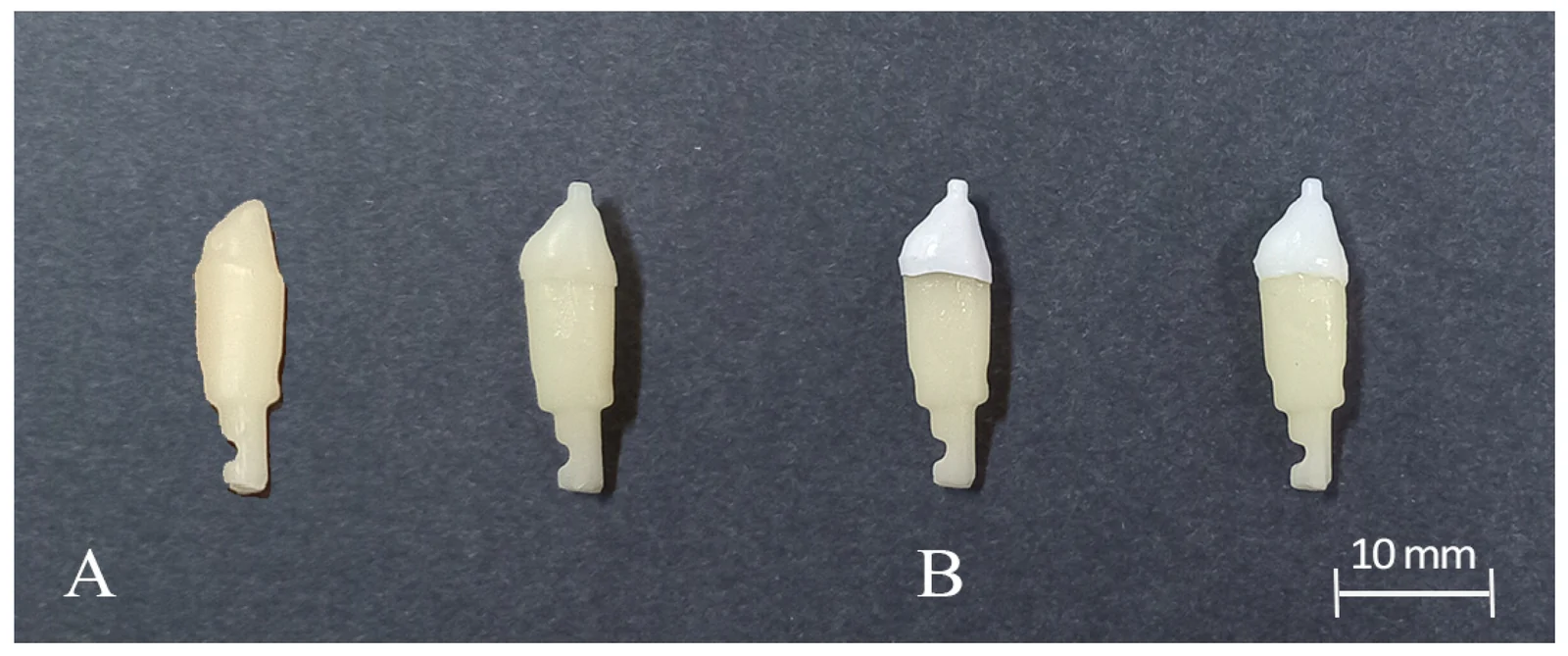
(**A**) Prepared mandibular artificial canine. (**B**) CAD-CAM fabricated copings with mini ball attachments for the three tested materials (PMMA, PEEK, zirconia).

An artificial acrylic mandibular canine (Banna study models and teeth, Alexandria, Egypt) was prepared to receive coping (Figure 1A). The preparation was scanned with an extraoral scanner (Activity 885, Smart optics Sensor technik GmbH, Bochum, Germany; accuracy 6 µm). The CAD design process followed a standardized protocol to ensure geometric consistency across all specimens. The mini ball attachment (1.7 mm diameter) selected from the exocad library corresponds to the Bredent Vario-Stud ball attachment system, featuring a spherical head dimension clinically validated for overdenture retention. The coping design incorporated a standard wall thickness of 0.8 mm with a 6° taper angle to ensure passive fit and consistent cement space. The cement gap was set at 40 μm to accommodate the luting agent while maintaining adequate retention. All 21 specimens were designed from the same digital file to eliminate design variability as a confounding factor.

Twenty-one identical replicas of the prepared canine were three dimensionally printed in PMMA. Using CAD software version 3.1 Rijeka (exocad GmbH, Darmstadt, Germany), we designed the copings and added mini ball attachments (1.7 mm diameter) from the Exocad library (Bredent GmbH& Co.KG, Senden, Germany) to the occlusal surface of each coping. The copings and attachments were divided into 3 groups, according to the material used for milling as follows: PEEK (breCAM BioHPP, bredent, Senden, Germany), Zirconia (discs with shoulder, Bettini Zirconia Dentale, Monte Marenzo, Italy), and PMMA (W-73015 PMMA Disc, SAGEMAX BIOCERAMICS, Federal Way, WA, USA) using a milling machine (DWX-51D, Roland, Shizuoka-ken, Japan). Post-milling, zirconia specimens underwent sintering in sintering furnace (TABEO-1/S/ZIRKON-100, MIHMVOGT, Baden-Württemberg, Germany) according to the manufacturer’s recommended protocol (Ramping to 1450 °C for 7 h and maintained at this temperature for 2 h and then normally cooled) to achieve full densification. 

The milled copings with attachments were cemented to the prepared canines using dual-cured resin cement (G-CEM, GC corporation, Tokyo, Japan) (Figure 1B). Before cementation, the fitting surface of each coping was sandblasted with 50 μm aluminum oxide for 10 s at a pressure of 2 bar. After sandblasting, specimens were subjected to ultrasonic cleaning in 70% isopropanol for 5 min. Excess cement was carefully removed, and light polymerization was performed through the coping for 20 s per surface (total 80 s) using a LED curing unit.

### 2.3. Retention Testing

Two plastic tubes were used for attaching the specimen to the universal testing machine (Instron, High Wycombe, UK). First, the tooth with its coping and attachment was fixed inside a plastic tube using a flowable light cured composite resin (Nexcomp flow, META BIOMED, Cheongju, South Korea); the tube was then clamped to the lower part of the testing machine. The second tube was then attached to the upper part of the machine by clamping. The nylon cap and metal housing were picked up by injecting light cured acrylic resin through small holes in the tube. It should be noted that the nylon caps were not replaced during the 2160 cycles. This decision was made to simulate the clinical scenario in which patients use the same attachment components over an extended period, with retention degradation occurring as a result of cumulative wear. In clinical practice, nylon caps are typically replaced only when retention drops below clinically acceptable levels, and the present study was designed to characterize the rate and pattern of retention decline under such conditions. This approach also allowed direct comparison of the wear patterns of the three attachment materials under identical conditions, without the confounding variable of cap replacement.

**Figure 2:**
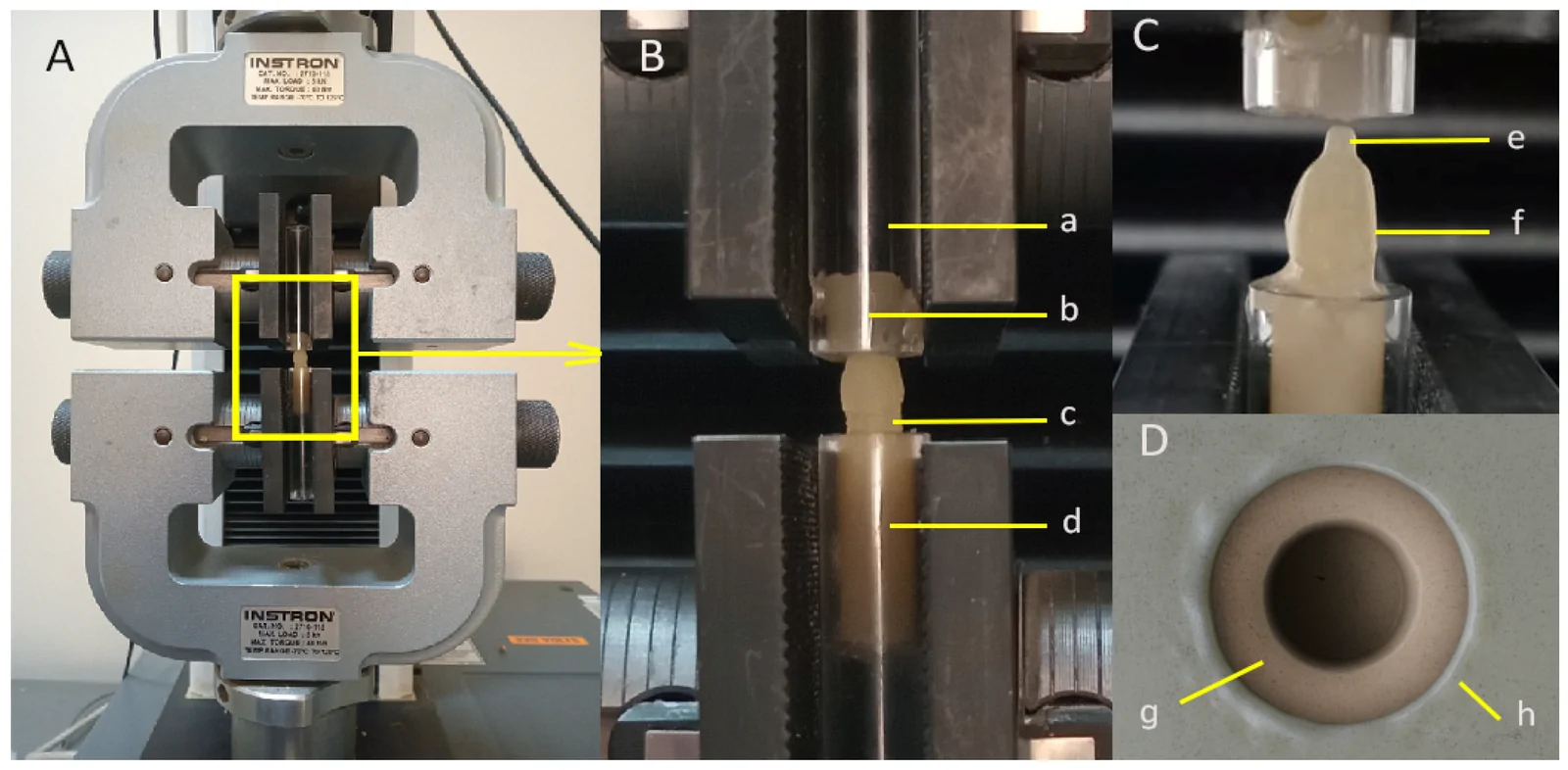
Experimental setup for retention testing. (**A**) Specimen mounted on the universal testing machine (yellow rectangle). (**B**) Specimen during pulling superior plastic tube (a) clamped to the upper machine member; light-cured composite resin (b) used to pick up the nylon cap and metal housing; artificial tooth (c) attached to the inferior plastic tube (d) with light-cured composite resin. (**C**) Specimen during pull-out, showing the attachment (e) and coping (f). (**D**) Inner surface of the superior plastic tube, revealing the nylon cap (g) and metal housing (h).

After the polymerization of composite resin, the upper arm of the machine moved at a cross-head speed of 5 mm/min. Retentive force was evaluated at baseline (T0) and after 90 cycles (T1), 270 cycles (T2), 540 cycles (T3), 1080 cycles (T4), and 2160 cycles (T5). One cycle was defined as complete insertion and removal, with manual cycling simulated patient use (three removals per day for two years). (Figure 2). To standardize the manual insertion–removal procedure and minimize operator-dependent variability, the following protocol was strictly followed: (1) All testing was performed by a single operator to eliminate inter-operator variability; (2) The operator was blinded to the material group assignment during the procedure; (3) The superior tube was lowered vertically at a controlled rate until complete seating was confirmed by both visual observation of full contact between the metal housing and the coping margin and tactile feedback of a “click” sensation; (4) The seated position was maintained for 1 s (timed with a digital metronome) to allow for nylon cap relaxation; (5) The tube was withdrawn vertically at a consistent speed through the measurement phase; (6) A rest interval of 30 s was maintained between cycles to allow for material recovery and minimize viscoelastic effects; (7) The testing environment was maintained at 23 ± 1 °C with 50 ± 5% relative humidity.

The universal testing machine (Instron 5960, High Wycombe, UK) was calibrated according to the manufacturer’s specifications prior to each testing session. A 100 N load cell, with an accuracy of ±0.5%, was employed. The cross-head speed (5 mm/min) was planned to simulate the typical rate of denture removal by patients while avoiding viscoelastic effects that could influence measured retention forces. The maximum tensile force recorded during separation was considered the retentive force for that cycle. Each measurement was repeated three times with 30 s intervals between repetitions to allow for nylon cap relaxation, and the mean value was recorded.

### 2.4. SEM Analysis

To explore the wear on the external surfaces of attachments, one representative specimen from each group was examined before and after final insertion–removal cycles using a field-emission scanning electron microscope (FE-SEM, Zeiss Sigma 500 VP, Jena, Germany). Samples were sputter-coated with gold–palladium (20 mA, 60 s). Surfaces were observed at magnification (×30) and at selected wear areas at magnification (×300) to assess wear patterns.

### 2.5. Statistical Analysis

Data were analyzed using SPSS version 28 (IBM Corp., Armonk, NY, USA). Normality was assessed using the Shapiro–Wilk test while homogeneity of variances was verified with Levene’s test. Descriptive statistics (mean, standard deviation) were calculated for each group at each time point. One way ANOVA was used to compare retentive forces among the three material groups at each of the six time points. Effect sizes (η^2^) were calculated for significant ANOVA results.

Within-group changes from baseline to final (2160 cycles) were analyzed using paired *t* tests with Bonferroni correction for three comparisons (adjusted α = 0.0167). Cohen’s d was reported as a measure of effect size. For paired *t*-tests, Cohen’s d was calculated using the pooled standard deviation formula to facilitate cross-study comparisons.

A mixed repeated measures ANOVA (between subjects factor: material; within subjects factor: time) was performed to evaluate the overall effect of time and the material × time interaction. Sphericity was assessed using Mauchly’s test, and the Greenhouse–Geisser correction was applied, with the epsilon (ε) value of 0.572 which reflects the degree of deviation from sphericity, when necessary. Partial η^2^ was reported for significant main effects. The use of partial eta-squared (η^2^p) as an effect size measure was selected for its suitability in repeated-measures designs, with values interpreted as small (0.01), medium (0.06), and large (0.14) effects.

All tests were two-tailed, and statistical significance was set at α = 0.05 unless otherwise specified.

## 3. Results

**Figure 3:**
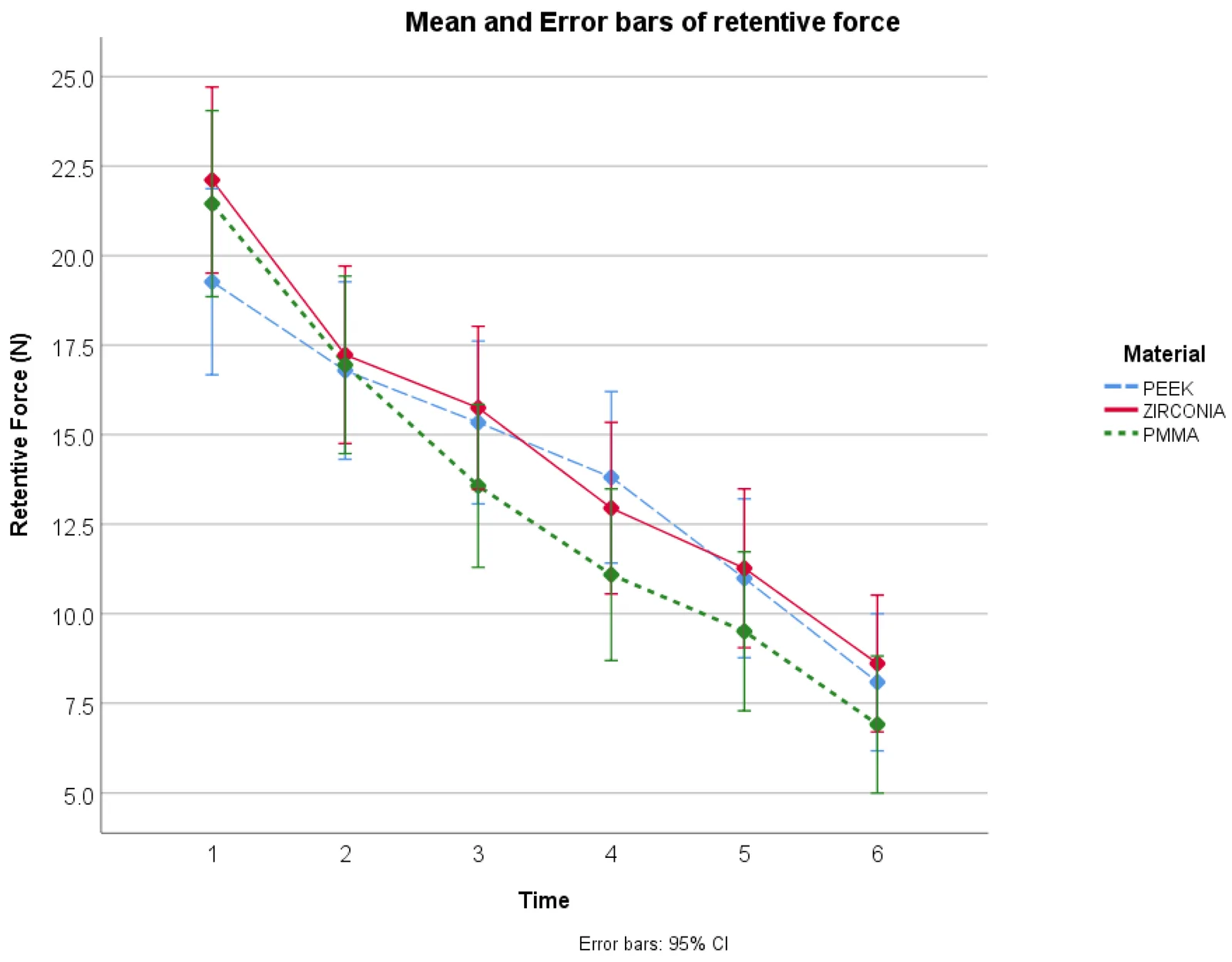
Profile plot of estimated marginal retentive forces (N) for PEEK, zirconia, and PMMA mini-ball attachments over 2160 insertion–removal cycles (T0 to T5). Error bars indicate 95% confidence intervals. The decline in retention over time was statistically significant (*p* < 0.001), whereas the material × time interaction was not (*p* = 0.064, Greenhouse–Geisser).

**Figure 4:**
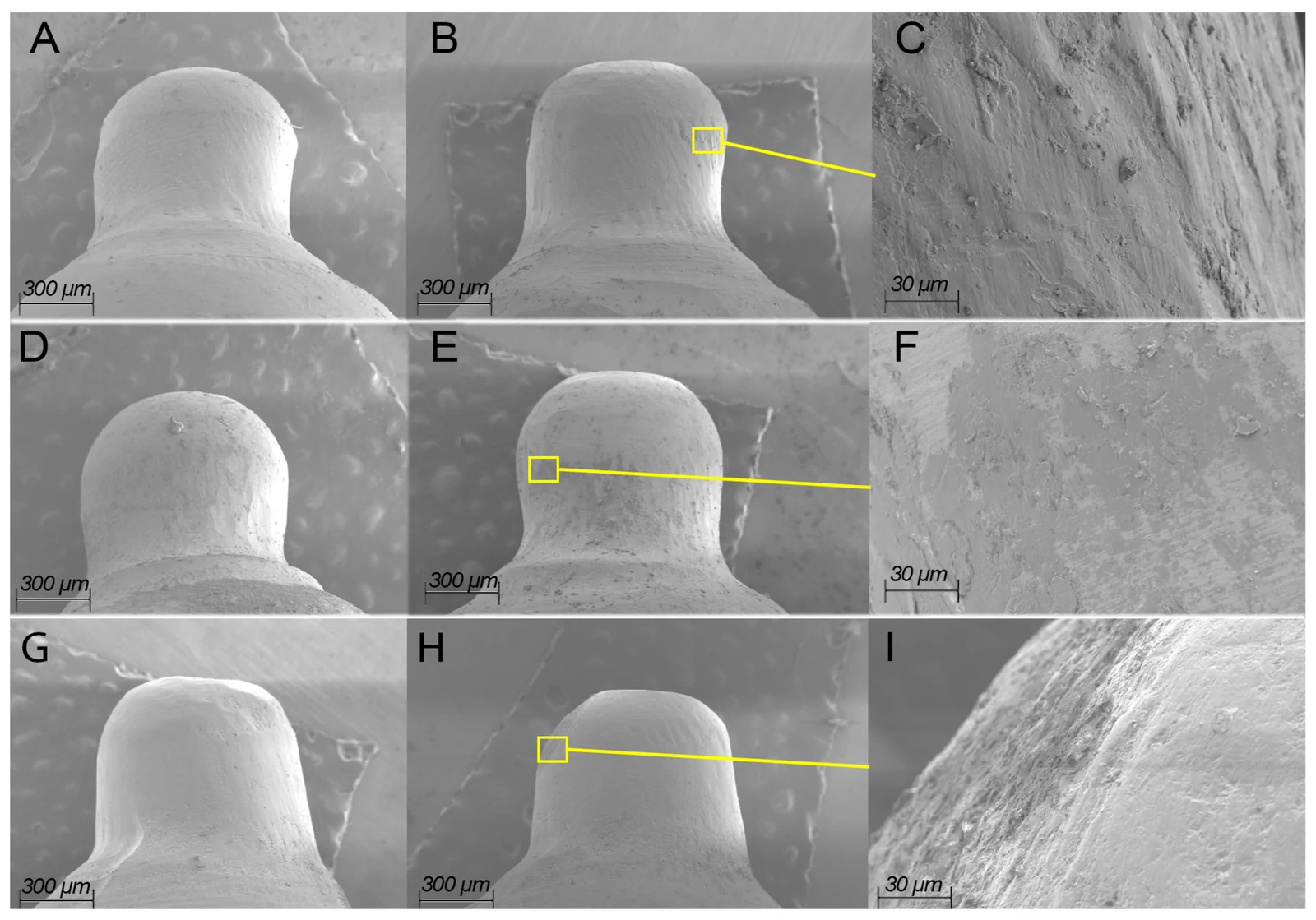
Representative scanning electron microscope images of mini ball attachments before and after cyclic loading (one specimen per material, preliminary observation). (**A**–**C**) PEEK: (**A**) before cyclic loading (×30), (**B**) after 2160 cycles (×30), (**C**) after 2160 cycles (×300). (**D**–**F**) Zirconia: (**D**) before cyclic loading (×30), (**E**) after 2160 cycles (×30), (**F**) after 2160 cycles (×300). (**G**–**I**) PMMA: (**G**) before cyclic loading (×30), (**H**) after 2160 cycles (×30), (**I**) after 2160 cycles (×300).

Descriptive statistics for retentive force at the six measurement time points are summarized in Table 1. Normality was confirmed for all combinations (*p* > 0.05) except for PMMA at T3 (*p* = 0.036) and zirconia at T5 (*p* = 0.048); however, given the robustness of parametric tests, ANOVA results were considered reliable. Levene’s test confirmed homogeneity of variances across groups at all time points (*p* > 0.05). One-way ANOVA revealed no statistically significant differences among materials at any time point (all *p* > 0.05; Table 1). A mixed repeated-measures ANOVA demonstrated a highly significant main effect of time (F(2.86, 34.31) = 168.55, *p* < 0.001, partial η^2^ = 0.90), indicating progressive retention loss over cycles. The material × time interaction was not significant (*p* = 0.064, partial η^2^ = 0.19), and the main effect of material was also non-significant (*p* = 0.584, partial η^2^ = 0.06). Pairwise comparisons for the main effect of time (Bonferroni adjusted) revealed that every time point differed significantly from every other (*p* < 0.001 for all comparisons). The mean retentive force declined from 20.95 N (SE = 0.71) at T0 to 7.88 N (SE = 0.53) at T5, a total reduction of 13.07 N (95% CI: 10.70 N to 15.44 N; Figure 3). Within-material comparisons (paired *t*-tests with Bonferroni correction, α = 0.0167) showed statistically significant reductions from baseline to final for all materials (*p* ≤ 0.009, Cohen’s d > 3.0; Table 2). Preliminary qualitative SEM revealed minimal surface changes for zirconia, moderate wear for PEEK, and pronounced surface deterioration for PMMA (Figure 4). It must be emphasized that these observations are purely descriptive, based on a single specimen per material, and are not statistically generalizable. No quantitative or comparative conclusions regarding material wear resistance can be drawn from these data.

## 4. Discussion

The selection of appropriate attachment materials for tooth-supported overdentures remains a clinical challenge, particularly as CAD/CAM technology expands the range of available non-metallic options. In this study, three non-metal materials (zirconia, PEEK, and PMMA) were tested as overdenture copings with mini ball attachments to examine how their retentive force changed over 2160 cycles. Surface wear was also explored using SEM to check changes in surface characteristics. The specimens of Zirconia, PEEK, and PMMA showed a decrease in the retentive force throughout the cyclic aging period. The results led us to reject the first null hypothesis, that retention would not decrease over time, because retention declined significantly for all three materials. The second null hypothesis (material type has no effect on retention or wear) could not be rejected for the retention data, although the wear analysis was only preliminary.

The mandibular canine tooth was selected because it is the most frequently retained last tooth, accounting for 63% of such cases, followed by the second molar (13%). Canine teeth are stable abutments for fixed or removable prostheses because of the length of the root and the anatomic shape of the crown, and the very little caries risk [14,15].

This study used a fully digital workflow, which stores the design in STL format. If a coping or attachment becomes damaged or loses retention, the stored STL data can be used to mill a new set without repeating the clinical or laboratory procedures [16].

Gurbulak et al. [17] and Schubert et al. [16] estimated artificial fatigue periods assuming that patients remove their dentures three times per day. Accordingly, 2160 cycles of artificial aging were selected to simulate an average period of 2 years of clinical use. PEEK, Zirconia, and PMMA groups recorded a gradual decrease in retention force through the artificial aging periods, which is a common phenomenon in the related literature. This decline is attributed to wear of the attachment components, loss of elasticity, and plastic deformation of the nylon caps [18].

Beyond mechanical performance and wear resistance, the biocompatibility of attachment materials represents a fundamental consideration in material selection. While zirconia, PEEK, and PMMA are generally considered biocompatible, their interaction with the oral soft tissues and the potential biological effects of wear debris warrant consideration.

At baseline, zirconia showed the highest mean retention and PEEK the lowest. At the final cycle, zirconia again showed the highest mean retention, while PMMA showed the lowest. Zirconia possesses high Vickers hardness (1200), high flexural strength (900–1200 MPa) and superior wear resistance compared to polymer-based materials. It resists surface abrasion and micro-cracking during repeated cycles, preserving the original fit and frictional force.

Zirconia was chosen because of its high performance as bar and telescopic attachments, although data on its use in ball attachments are scarce. Zirconia bars have been associated with high patient satisfaction, shorter fabrication times, good hygiene, and high clinical reliability with minimal maintenance [19,20]. Zirconia primary telescopic crowns showed high wear resistance and stable retention [21,22,23,24]. PEEK exhibited lower retention than zirconia, consistent with its much lower hardness (20–30 HV vs. 1200 HV for zirconia). Under cyclic stress, this makes PEEK more susceptible to surface wear and deformation. The use of CAD/CAM for the construction of zirconia attachment produces less stresses when compared with metal attachment constructed using the casting technique [25]. Abd Allah et al. [26] reported less stresses with extracoronal attachments constructed from zirconia material than their counterparts constructed from PEEK material.

The literature reports several recommended retention force ranges per abutment to stabilize removable prostheses without traumatizing the supporting teeth or implants: 5–7 N [27], 5–9 N [28], 5–10 N [29], and 2.5–6.5 N [30]. Within the constraints of this laboratory test model, the final retention values (7.06–8.76 N) fell within the ranges reported as potentially adequate for removable prostheses (e.g., 5–10 N). However, several cautions must be considered. First, direct clinical extrapolation is not justified because the oral environment was not fully simulated, thermocycling, chemical ageing, and masticatory forces were absent. Second, the retention ranges cited in the literature derive from diverse attachment systems and testing methodologies, and the optimal retention value may be system-specific. Third, retention requirements vary based on individual patient factors like ridge morphology, mucosal resilience, and functional needs. Fourth, not only retention is important for prosthetic success; stability, support, and patient comfort are equally important. While the measured retention values are promising, they should be interpreted as preliminary evidence of the potential feasibility of these materials, rather than as a definitive indication of clinical adequacy. The results suggest that these materials merit further investigation under more clinically relevant conditions.

In the wear assessment, zirconia showed the least surface change, probably because of its highly polished, smooth surface, which reduces friction and abrasive wear. PEEK, with much lower hardness (20–30 HV), was more vulnerable to surface deterioration and showed moderate wear. The low hardness of PMMA also makes it extremely susceptible to surface wear and scratching under repeated friction [31]. Under cyclic loading in resin-based materials [32], microcracks can develop and propagate, leading to surface roughening and deep scratches, as observed in our SEM images.

This study has limitations. First, the wear analysis was performed on only one specimen per material using SEM as an exploratory measure. The differences observed are therefore qualitative and illustrative, not quantitative or comparative. This limitation must be emphasized: the SEM findings are not statistically generalizable and should not be interpreted as evidence of superior or inferior wear resistance of any material. Future studies with larger samples and quantitative wear assessment methodologies are required to establish definitive comparative wear data. Second, the insertion–removal cycles were performed manually, introducing potential variability in the angle, speed, and force of separation. Third, and critically, the experimental conditions did not fully replicate the oral environment. The absence of thermocycling (which exposes materials to thermal changes ranging from 5 °C to 55 °C by hot and cold beverages) may have underestimated the degradation of retention over time. Similarly, the lack of chemical ageing by artificial saliva, acidic challenges, or food debris may have influenced wear patterns. The cyclic loading protocol also did not simulate the complex masticatory cycle (including lateral and rotational movements), potentially affecting the mode and rate of wear. Additionally, the testing was performed under dry conditions rather than in a hydrated state, which may have influenced the tribological properties of PEEK and PMMA in particular. Given these limitations, the findings should be interpreted with caution, but they may provide a basis for future well-powered, clinically simulated investigations.

### Future Research Directions

The findings of this study suggest several promising avenues for future investigation. First, quantitative wear analysis using three-dimensional profilometry or optical coherence tomography would provide objective measurements of surface degradation and enable correlation with retention decline. Second, the effects of artificial saliva and pellicle formation on attachment tribology merit investigation, as these biological factors significantly influence friction coefficients and wear behavior in vivo. Third, the potential for surface modifications, such as the application of diamond like carbon coatings or plasma surface treatments, to enhance the wear resistance of polymer-based attachments should be explored. Fourth, the development of hybrid attachments combining ceramic surfaces with polymeric matrices may offer an optimal balance of wear resistance and stress absorption. Fifth, long-term clinical trials with larger patient populations are essential to confirm the laboratory findings and establish evidence-based recommendations for material selection. Additionally, economic analysis comparing the cost effectiveness of these materials in clinical practice would inform health economic decision making. Finally, given the preliminary nature of the SEM findings, future studies should incorporate quantitative wear assessment with larger sample sizes to enable statistical comparison of wear resistance among materials.

## 5. Conclusions

Within the limitations of this laboratory study, the following can be concluded:

1. All three CAD CAM fabricated mini ball attachments (zirconia, PEEK, PMMA) showed a significant and substantial decline in retentive force over 2160 insertion removal cycles. The effect of time was large (partial η^2^ = 0.90).
2. No statistically significant differences in retentive force were observed among the three materials at any measurement time point. The material versus time interaction was also non-significant, indicating similar rates of retention loss.
3. Preliminary qualitative SEM suggested that PMMA exhibited the most surface wear, whereas zirconia showed the least. These observations should be interpreted with caution and require quantitative confirmation.
4. Study findings need to be confirmed in future clinical investigations.

## Figures and Tables

**Table 1:** Retentive force (N) at each measurement cycle for PEEK, zirconia and PMMA mini-ball attachments, with one-way ANOVA results.

Material	T0	T1	T2	T3	T4	T5
PEEK	19.28 ± 3.73	16.80 ± 3.42	15.35 ± 2.56	13.82 ± 2.27	11.00 ± 2.64	8.10 ± 2.55
Zirconia	22.12 ± 3.20	17.24 ± 1.46	15.76 ± 1.60	12.96 ± 2.35	11.28 ± 1.88	8.62 ± 2.38
PMMA	21.46 ± 2.83	16.96 ± 3.92	13.58 ± 3.94	11.10 ± 4.07	9.52 ± 3.59	6.92 ± 2.28
ANOVA *p*-value	0.262	0.965	0.340	0.253	0.464	0.417

Data are presented as mean ± standard deviation (n = 7 per group). No significant differences were found among materials at any time point (one way ANOVA, *p* > 0.05).

**Table 2:** Within-material comparison of retentive force (N) between baseline (T0) and final (T5) cycles.

Material	Baseline (T0)	Final (T5)	Mean Difference (T0–T5)	95% CI of Difference	*p*-Value *	Cohen’s d
PEEK	19.28 ± 3.73	8.10 ± 2.55	11.18	7.20–15.16	0.009	3.50
Zirconia	22.12 ± 3.20	8.62 ± 2.38	13.50	10.86–16.14	0.001	4.60
PMMA	21.46 ± 2.83	6.92 ± 2.28	14.54	12.62–16.46	<0.001	5.70

* Paired *t*-test with Bonferroni correction (α = 0.0167). All differences are statistically significant. Cohen’s d calculated from pooled standard deviations.

## Data Availability

The original contributions presented in this study are included in the article. Further inquiries can be directed to the corresponding authors.

## Abbreviations

The following abbreviations are used in this manuscript:

PEEK	Polyetheretherketone
PMMA	Polymethyl methacrylate
CAD CAM	Computer-aided design computer aided-manufacturing
SEM	Scanning electron microscopy
